# Relationship between changes in the public health nurses’ workforce and the empirical Bayes estimates of standardized mortality ratio: a longitudinal ecological study of municipalities in Japan

**DOI:** 10.1186/s12913-023-09273-2

**Published:** 2023-03-17

**Authors:** Shimpei Kodama, Futoshi Uwatoko, Chihaya Koriyama

**Affiliations:** 1 Department of Comprehensive Community-Based Nursing Science, School of Health Sciences, Faculty of Medicine, Kagoshima University, 8-35-1 Sakuragaoka, Kagoshima, 890-8544 Japan; 2 Department of Epidemiology and Preventive Medicine, Graduate School of Medical and Dental Sciences, Kagoshima University, Kagoshima, Japan

**Keywords:** Public health nurse, Workforce, Municipalities, Japan, Empirical Bayes estimate of standardized mortality ratio

## Abstract

**Background:**

The role of public health nurses (PHNs) in the community is expected to become increasingly important, along with the promotion of a comprehensive community care system. However, a comprehensive study of all municipalities is yet to be undertaken, and the relationship between the workforce of PHNs and health indicators is yet to be clarified. This study examined the effect of workforce change among PHNs, one of the structural indicators of PHNs’ activities regarding changes in the empirical Bayes estimate of standardized mortality ratios (EBSMRs).

**Methods:**

An ecological study was conducted using municipality-level aggregate data. The data used were publicly available Japanese government statistics. The first-difference model of panel data analysis was used to examine the relationship between changes in EBSMR and changes in the number of PHNs per 100,000 population from 2010 to 2015, adjusting for the effects of population and other healthcare resources, including the number of physicians, medical clinics, general hospitals, and welfare facilities. The variation by the 47 prefectures was added to the linear model as a random effect. We also performed a sensitivity analysis using the full Bayesian inference using the Besag-York-Mollie model.

**Results:**

For males, EBSMRs for all causes and malignant neoplasms significantly decreased with an increase in the number of PHNs per population (coefficients: -1.00 and -0.89, *p* values: 0.008 and 0.043, respectively). For females, although all EBSMRs except malignant neoplasms showed decreased tendencies due to the increase in the number of PHNs per population, none of them were significant. The full Bayesian inference confirmed these associations.

**Conclusions:**

An increase in the number of PHNs per population was significantly associated with a greater reduction in deaths from all causes and malignant neoplasms in males. The results of the full Bayesian inference also suggest that the workforce of PHNs may be related to changes in standardized mortality ratios for deaths from all causes in females.

**Supplementary Information:**

The online version contains supplementary material available at 10.1186/s12913-023-09273-2.

## Background

Increasing healthcare expenditures in Japan due to the declining birthrate and aging population necessitates the efficient allocation of healthcare resources while balancing their objectives of cost control and quality improvement [1]. Regional variation in the contexts surrounding these healthcare settings [2] affects local health systems and health outcomes [3–5]. Public health nurses (PHNs) primarily address these regional characteristics as they perform healthcare activities.

A PHN in Japan requires a national certification different from that of a registered nurse. Moreover, many PHNs work as civil servants in prefectures and municipalities. As of 2018, approximately 70% of the 53,000 PHNs in Japan were civil servants [6]. PHNs provide various community care services for both individuals and the community as a whole. For example, in addition to interpersonal services, they provide comprehensive community healthcare and policy recommendations to help aging community members live independently [7–9]. In recent years, most Japanese public health interpersonal services are shifting from prefectures to municipalities, including cities, towns, and villages. As a result, the number of PHNs working in prefectures has decreased, and the number of PHNs working in municipalities has increased [10]. Therefore, the role of PHNs in municipalities is becoming increasingly important in building an effective community healthcare system in Japan.

Considering the activities of Japanese PHNs as civil servants, it is crucial to evaluate the effectiveness of their health and welfare activities, and to link them to the formulation of municipal policies. The evaluation of healthcare quality is often explained in the framework of the relationship between structure (e.g., the quantity of healthcare resources and workforce), process (e.g., the content of activities), and outcomes (e.g., health indicators) presented by Donabedian [11]. In Japan, as local governments' finances are deteriorating, a structure-focused evaluation is crucial for appropriate allocation of healthcare resources. However, despite the literature examining the relationship between healthcare resources and outcomes in municipalities [12], studies focusing on the impact of the PHN workforce on outcomes—a structural indicator directly related to labor costs—have yet to provide consistent evidence [13–17]. In addition, most existing studies were conducted at the prefectural or district level [13–16], and only one study was conducted for municipalities, albeit in a single prefecture [17]. For assessing the quality of health care in Japan, it is important to examine the activities of PHNs at the municipal level, as these account for the majority of interpersonal public health services availed in Japan. Therefore, we conducted a study in 2019 examining municipalities across Japan to determine the impact of the PHN workforce on standardized mortality ratios (SMRs), and we found that the workforce of PHNs, especially part-time PHNs, is associated with low SMR [18]. This study, however, focused on municipalities with a population of 10,000 or more for which reliable SMRs could be calculated, and it was cross-sectional. A comprehensive study of all municipalities, including those with populations under 10,000, is yet to be undertaken, and the relationship between changes in the workforce of PHNs and changes in SMRs, contributing to more critical evidence for policymaking, is yet to be clarified.

Given the aging population, the prevalence of rising chronic diseases, and increasing social complexity, strengthening community healthcare systems worldwide has become a major focus for healthcare improvement, and the role of the PHN has become integral to this endeavor [19]. Examining the effect of workforce changes among municipal PHNs in Japan, a nation with one of the lowest birthrates and the highest proportion of the aging population in the world, will benefit future policy decisions across countries.

This study aimed to longitudinally examine the effect of workforce changes among PHN in municipalities across Japan using changes in Bayesian estimates of SMRs as outcomes based on municipal data for 2010 and 2015 released by the government.

## Methods

### Data sources

Data were collected from the government statistics portal e-Stat [20] and included the Population Census, the Specific Report of Vital Statistics, the Report on Regional Public Health Services and Health Promotion Services, the Survey of Physicians, Dentists, and Pharmacists, the Dynamic Survey of Medical Institutions, and the Survey of Institutions and Establishments for Long-term Care. The census is from the Statistics Bureau of the Ministry of Internal Affairs and Communications, and the other data are from the Ministry of Health, Labour, and Welfare (MHLW). Data were analyzed for the years 2010 and 2015 (census survey years) or the nearest year. The two survey years were selected because the statistics on deaths in each municipality are published every five years; furthermore, the analysis focuses on years after 2010, as large-scale municipal mergers took place in Japan between 1999 and 2006, making it difficult to compare data from earlier years.

### Study sample

The study sample comprises the municipalities in Japan listed in the latest Specific Report of Vital Statistics in FY2013-FY2017 [20]. We excluded 92 municipalities (including special wards in Tokyo) from the 1,741 municipalities listed in this report because they were “health center–designated cities” where health services of prefectures are exceptionally provided by municipalities, and therefore the assignment and working style of PHNs are considered different from those of ordinary municipalities. We also excluded 48 municipalities that did not have data for either 2010 or 2015 due to the Great East Japan Earthquake; consequently, 1,601 municipalities were included in the analysis.

### Outcomes

The outcomes of this study focused on SMRs for all causes of death and the three leading causes of death in Japan; malignant neoplasms (C00-C97), heart disease (I01-I02.0, I05-I09, I20-I25, I27, I30-I51), and cerebrovascular disease (I60-I69). By calculating the empirical Bayes estimate of SMR (EBSMR), which is one of the Bayesian methods to smoothen SMR values by the spatial similarity of the surrounding areas [21], this study eliminates the limitations of standard SMR for small municipalities, where SMR variability is high, and results are difficult to interpret [22]. We used the moment estimates recommended in existing studies for the estimation [23]. The prior distribution of SMRs was determined from the distribution of SMRs per prefecture. To smooth out variation across years, we used the combined five-year death count data to calculate the EBSMR (data from 2008–2012 and 2013–2017 were used for the 2010 EBSMR and 2015 EBSMR, respectively). In the case of using the indirect standardization method, it is technically appropriate to compare EBSMR only with the rate of the standard population. Therefore, to allow for longitudinal comparisons, the 2010 national age-specific mortality rates by five-year age groups were used as the standard in the calculation of EBSMRs for both 2010 and 2015. All EBSMRs in this study are based on the 2010 national value of 100. Since the incidence of diseases differs by gender and the original data are separated by gender, EBSMRs were calculated separately for males and females. The EBSMRs were calculated using the empirical Bayes (EB) estimator for Poisson-Gamma model version 2.1 [24]. The EB estimator calculates EBSMR using the Poisson distribution as the probability distribution followed by the actual number of deaths and the gamma distribution as the prior and posterior distributions followed by the standardized death ratio.

### Influence of healthcare resources on outcomes

The primary influencing factor was the number of PHNs per 100,000 population. Because our previous study has shown that the PHN workforce, especially part-time PHNs, was associated with low SMRs [18], in addition to the full-time PHNs, the part-time PHNs were converted to full-time and added to the number of PHNs. Following the previous study, we used the number of physicians, the number of medical clinics, the number of general hospitals in a secondary healthcare area to which the municipality belongs, the number of welfare facilities for the elderly requiring long-term care (all per 100,000 population), and population as adjustment factors affecting the relationship between the number of PHNs and outcomes [18]. The number of nurses per population was not included in the adjustment factor because of its strong relationship with the number of medical clinics and general hospitals. For longitudinal comparisons, the population of 2010 was used to calculate per 100,000 population for both 2010 and 2015.

### Statistical analysis

This study uses panel data from two-time points. The analysis of panel data typically uses the fixed-effects model, which has an advantage in the ability to control for unobserved bias due to constant factors throughout the study period [25, 26]. This ability is well suited to this study, an ecological study with many unobserved factors. However, a typical fixed-effects model does not consider sequential effects between time points. Therefore, this study adopted the first-difference model, which analyzes the difference between two-time points to examine the time-series–based relationship between outcomes and influencing factors. The estimators of the first-difference model and the fixed-effects model are numerically equal, while the first-difference model calculates coefficients that take into account the time series. [25].

To examine the first-difference model, we conducted an analysis via the maximum likelihood estimation method, using a no-intercept linear model with changes in EBSMR as the dependent variable and changes in the number of PHNs per 100,000 population and the other healthcare resources as independent variables. The population distribution by the municipality in Japan was fitted with a log-normal distribution model [27], and municipalities with small populations had more PHNs per population than those with large populations [28]. Therefore, for the population and the number of PHNs per 100,000 population, a logarithmic transformation was performed on the natural logarithm, and the difference was calculated and included in the model. Additionally, because the status of deaths in a region varies by prefecture [29], and the temporal effects of unobserved factors in each prefecture may affect SMRs, we added the variation by the 47 prefectures to the linear model as a random effect. After the examination of the first-difference model using all PHNs for the entire municipality, sensitivity analyses were conducted using models with full-time and part-time PHNs instead of all PHNs. We also conducted analyses stratified by the population at a baseline of more than or less than 10,000 and stratified by EBSMRs at a baseline of less than or more than 100 for each cause.

The first-difference model can simplify the relationship between changes in EBSMRs and changes in the number of health resources comprehensively. However, since the amounts of medical resources were also an important factor for SMRs in our previous study [18], it is vital for this study to confirm the relationship between SMRs and the number of PHNs, with a more detailed adjustment for the amounts of medical resources at each time point. Therefore, for further sensitivity analysis, we applied the Besag-York-Mollie (BYM) model [30–32], a hierarchical Bayesian estimation method for relative risk using Poisson distribution, to examine the effect of the change of the number of PHNs on the number of deaths. For spatial smoothing, the BYM model uses the intrinsic conditional autoregressive (ICAR) component, which is smoothed by the average relative risk of neighboring regions, while the EBSMR was smoothed based on prefectural information used in government statistics and is intuitively understandable. By using the ICAR model with full Bayesian inference, the BYM model can provide more statistically reliable parameter estimates.

As the BYM model examines the effect on the number of deaths at each time point, it cannot directly examine the effect on changes in the number of deaths. Therefore, the BYM model analysis first examined the relationship between the number of deaths and the number of PHNs using data from both time points. Then, to examine the impact of changes in the number of PHNs on this relationship, a similar analysis was conducted with the PHN variable split into the baseline number of PHNs in 2010 and the change in the number of PHNs from 2010 to 2015. The BYM model used the same adjustment factors as the first-difference model (the number of physicians, medical clinics, general hospitals in a secondary healthcare area to which the municipality belongs, and welfare facilities for older adults requiring long-term care per 100,000 population) and population. In addition, a time dummy variable equal to 0 for 2010 and 1 for 2015 representing the change in the dependent variable over time and an offset term of the log-transformed expected number of deaths in each municipality were added to the independent variables. The BYM model in this study can be described as follows.

$${o}_{i} \sim \mathrm{Poisson }\left({\mu }_{i}\right)$$

$$\mathrm{log}\left({\mu }_{i}\right)=\mathrm{log}\left({e}_{i}\right)+{{\varvec{x}}}_{i}\upbeta +{\mathrm{\varphi }}_{i}+{\upepsilon }_{i}$$

where $o$ is the observed number of deaths in each municipality. $e$ is the number of deaths expected from the age-specific reference mortality rates. The SMR is $o/e$. $\mu$ is the expected value of $o$, and if the expected value of the SMR is ${\uptheta }_{i}$, then ${\mu }_{i}={e}_{i}{\uptheta }_{i}$, and the $\mathrm{log}\left({e}_{i}\right)$ is treated as an offset term. ${\varvec{x}}$ is the matrix of independent explanatory variables, and $\upbeta$ is the vector of regression coefficients. $\mathrm{\varphi }$ is the ICAR component for special smoothing, and $\upepsilon$ is a component for non-spatial heterogeneity. Further details on the BYM model and full Bayesian inference in this study are provided in Additional file 1.

Prior to the analysis of associations using models adjusting for healthcare resources other than PHNs, descriptive statistics and bivariate associations were performed for all municipalities, and to confirm the characteristics of small municipalities, municipalities with a population of less than and more than 10,000 as of 2010 were examined separately. Each analysis was conducted according to gender. IBM SPSS Statistics 27 [33] was used for the analysis other than hierarchical Bayesian estimation, and the rstan package version 2.21.2 [34] of R version 4.1.2 [35] with RStudio (2021.09.1 + 372) [36] was used for the analysis of hierarchical Bayesian estimation. The significance level was set at 0.05.

## Results

The baseline (2010) descriptive statistics for EBSMR with crude death rate (CDR) as a reference and healthcare resources are shown in Table 1, and the changes in the respective data until 2015 are shown in Table 2. As of 2010, there were 470 municipalities with a population of fewer than 10,000 people and 1,131 municipalities with more than 10,000 people. The lowest EBSMR was 95.6 for malignant neoplasms in females. There were large differences in healthcare resources, including the number of PHNs per 100,000 population, depending on the size of the municipality (absolute values of Cohen’s d > 1.0). In contrast, the differences in EBSMRs by municipality size were relatively small, all less than medium (absolute value of Cohen's d < 0.5). Changes until 2015 show an overall downward trend in EBSMRs and an upward trend for the number of health professionals per 100,000 population.

**Table 1:** Descriptive statistics (2010)

	All municipalities		Population < 10,000		Population ≥ 10,000		Cohen's d
	(*n* = 1601)		(*n* = 470)		(*n* = 1131)		
	Mean	(SD)	Mean	(SD)	Mean	(SD)	
EBSMRs in males							
All causes of death	101.0	(8.7)	101.9	(9.0)	100.6	(8.5)	0.15
Malignant neoplasms	98.7	(8.8)	99.0	(10.0)	98.5	(8.2)	0.06
Heart disease	102.1	(16.9)	102.7	(18.6)	101.8	(16.1)	0.05
Cerebrovascular disease	103.4	(21.0)	103.8	(23.0)	103.3	(20.1)	0.03
EBSMRs in females							
All causes of death	99.6	(9.2)	98.6	(10.7)	100.0	(8.4)	-0.15
Malignant neoplasms	95.6	(9.3)	93.8	(11.4)	96.4	(8.1)	-0.28
Heart disease	101.2	(17.0)	99.6	(19.5)	101.9	(15.9)	-0.14
Cerebrovascular disease	102.9	(22.3)	100.8	(24.8)	103.8	(21.1)	-0.14
CDRs in males							
All causes of death^a^	3216.4	(960.6)	3975.5	(967.1)	2901.0	(761.0)	1.30
Malignant neoplasms^a^	1014.3	(296.3)	1223.7	(361.7)	927.3	(210.2)	1.12
Heart disease^a^	460.8	(184.2)	563.3	(237.0)	418.1	(136.1)	0.84
Cerebrovascular disease^a^	317.7	(151.6)	391.8	(211.0)	286.9	(104.1)	0.73
CDRs in females							
All causes of death^a^	2983.1	(1017.4)	3675.1	(1103.4)	2695.5	(823.7)	1.07
Malignant neoplasms^a^	665.7	(207.1)	770.1	(268.3)	622.3	(156.3)	0.75
Heart disease^a^	551.3	(245.8)	680.2	(318.1)	497.7	(183.7)	0.79
Cerebrovascular disease^a^	354.9	(172.6)	428.7	(227.6)	324.2	(132.1)	0.63
The number of healthcare resources							
PHNs^a^	43.2	(41.8)	84.8	(55.2)	25.9	(13.5)	1.84
Full-time PHNs^a^	41.9	(41.4)	82.7	(55.2)	24.9	(13.4)	1.81
Part-time PHNs^a^	1.3	(4.0)	2.1	(6.8)	1.0	(1.8)	0.28
Physicians^a^	144.2	(141.2)	86.0	(66.7)	168.4	(156.2)	-0.61
Medical clinics^a^	73.0	(49.1)	87.4	(83.4)	67.0	(20.1)	0.42
General hospitals in a secondary healthcare area^a^	7.2	(3.5)	8.2	(3.7)	6.8	(3.3)	0.41
Welfare facilities for the elderly requiring long-term care^a^	9.5	(10.5)	16.6	(16.3)	6.5	(4.1)	1.06
Population	44,299.2	(57,711.8)	5093.0	(2627.7)	60,591.8	(61,709.6)	-1.07

CDR, crude death rate; EBSMR, empirical Bayes estimate of standardized mortality ratio; PHN, public health nurse

^a^ Per 100,000 population

**Table 2:** Change of EBSMRs and the number of healthcare resources (2015–2010)

Variables of change (2015–2010)	All municipalities		Population < 10,000		Population ≥ 10,000		Cohen's d
	(*n* = 1601)		(*n* = 470)		(*n* = 1131)		
	Mean	(SD)	Mean	(SD)	Mean	(SD)	
EBSMRs in males							
All causes of death	-7.8	(5.6)	-7.6	(7.2)	-7.8	(4.9)	0.04
Malignant neoplasms	-7.1	(6.6)	-6.8	(8.7)	-7.2	(5.5)	0.06
Heart disease in males	-10.7	(13.0)	-10.4	(18.1)	-10.9	(10.1)	0.04
Cerebrovascular disease	-23.1	(16.2)	-23.0	(21.4)	-23.1	(13.5)	0.01
EBSMRs in females							
All causes of death	-3.7	(7.0)	-2.4	(9.7)	-4.3	(5.4)	0.27
Malignant neoplasms	-2.6	(8.1)	-1.9	(11.4)	-2.9	(6.3)	0.12
Heart disease	-10.4	(13.3)	-8.8	(18.3)	-11.0	(10.5)	0.17
Cerebrovascular disease	-22.0	(16.8)	-20.3	(23.0)	-22.7	(13.3)	0.15
Number of healthcare resources							
PHNs ^a^	2.8	(22.2)	6.6	(38.9)	1.2	(7.8)	0.24
Full-time PHNs ^a^	2.4	(22.1)	5.9	(38.8)	0.9	(7.6)	0.23
Part-time PHNs ^a^	0.4	(5.2)	0.6	(9.1)	0.3	(2.1)	0.06
Physicians ^a^	2.1	(27.8)	-3.9	(36.1)	4.6	(23.0)	-0.31
Medical clinics ^a^	0.2	(10.4)	-0.3	(16.3)	0.4	(6.5)	-0.07
General hospitals in a secondary healthcare area ^a^	-0.3	(0.6)	-0.4	(0.7)	-0.2	(0.5)	-0.27
Welfare facilities for the elderly requiring long-term care ^a^	1.5	(6.6)	1.7	(11.3)	1.4	(2.9)	0.05
Population	-883.7	(2040.6)	-387.5	(308.9)	-1089.9	(2389.9)	-1.08

EBSMR, empirical Bayes estimate of standardized mortality ratio; PHN, public health nurse

^a^ Per 100,000 population

Simple regression analysis without an intercept was conducted on the bivariate association between the change in EBSMRs and the change in the number of PHNs per 100,000 population (Table 3). There were significant negative associations for all causes of death and all three major diseases, except for malignant neoplasms in females. Similar negative associations were also observed in analyses with baseline populations of less than and more than 10,000, although some associations were not statistically significant.

**Table 3:** Bivariate association between changes in EBSMRs and PHNs by no-intercept simple regression analysis

Variables of change (2015–2010)	All municipalities		Population < 10,000		Population ≥ 10,000	
	(*n* = 1601)		(*n* = 470)		(*n* = 1131)	
	Coef^b^	*P-value*	Coef^b^	*P-value*	Coef^b^	*P-value*
EBSMRs in males^a^						
All causes of death	-4.42	< 0.001	-3.39	0.001	-5.30	< 0.001
Malignant neoplasms	-4.02	< 0.001	-3.86	< 0.001	-4.16	< 0.001
Heart disease in males	-3.65	0.002	-1.02	0.630	-5.88	< 0.001
Cerebrovascular disease	-11.60	< 0.001	-8.20	0.009	-14.49	< 0.001
EBSMRs in females^a^						
All causes of death	-2.08	< 0.001	-1.61	0.111	-2.48	< 0.001
Malignant neoplasms	-0.49	0.405	-0.39	0.739	-0.57	0.375
Heart disease	-5.06	< 0.001	-3.49	0.089	-6.40	< 0.001
Cerebrovascular disease	-10.99	< 0.001	-7.62	0.014	-13.87	< 0.001

*Coef* Coefficient, *EBSMR* Empirical Bayes estimate of standardized mortality ratio, *PHN* Public health nurse

^a^ Dependent variables

^b^ Coefficients of PHNs (2015–2010) per 100,000 population (logarithmic transformed)

The analysis results using the no-intercept linear model, which included changes in the other adjustment factors, are shown in Table 4. A larger increase in the number of PHNs per 100,000 population resulted in a larger decrease in EBSMR for all causes of death and malignant neoplasms in males (coefficient = -1.00, *p* = 0.008 and coefficient = -0.89, *p* = 0.043, respectively). For females, no EBSMR was significantly associated with a change in the number of PHNs though all EBSMRs except malignant neoplasms showed decreased tendencies due to the increase in the number of PHNs per population. In all the linear models, Akaike's information criterion (AIC) was lower than that of the model without the random effect (see table in Additional file 2: Table S1), indicating that the input of the differences by prefecture improved the fit of the model. In addition, the variance inflation factors (VIFs) among the independent variables other than the random effect ranged from 1.01 to 1.12. This suggests that multicollinearity did not affect the results.

**Table 4:** Relationship between changes in EBSMRs and PHNs (*n* = 1601)

Variables of change (2015–2010)	**Changes in PHNs (2015–2010)**						
	**All PHN model**			**Full-time and part-time PHN model**			
	All PHNs^b^			Full-time PHNs^b^		Part-time PHNs^b^	
	Coef^c^	*P-value*	[AIC]	Coef^c^	*P-value*	Coef^c^	*P-value*
EBSMRs in males^a^							
All causes of death	-1.00	0.008	[10135.1]	-0.98	0.005	0.06	0.727
Malignant neoplasms	-0.89	0.043	[10614.9]	-0.85	0.038	-0.18	0.403
Heart disease in males	0.79	0.354	[12716.1]	0.46	0.558	-0.15	0.728
Cerebrovascular disease	-0.97	0.373	[13511.8]	-0.81	0.417	1.042	0.050
EBSMRs in females^a^							
All causes of death	-0.38	0.416	[10749.3]	-0.06	0.894	0.06	0.778
Malignant neoplasms	0.71	0.194	[11211.7]	1.32	0.009	-0.35	0.188
Heart disease	-0.76	0.405	[12902.8]	-0.74	0.377	-0.04	0.930
Cerebrovascular disease	-1.11	0.329	[13645.6]	-0.41	0.698	0.70	0.206

*AIC* Akaike's information criterion, *Coef* Coefficient, *EBSMR* Empirical Bayes estimate of standardized mortality ratio, *PHN* Public health nurse

^a^ Dependent variables

^b^ Per 100,000 population (logarithmic transformed)

^c^ Coefficients are adjusted for random effects by prefecture and fixed effects by change of the number of population (logarithmic transformed) and healthcare resources per 100,000 population (2015–2010): physicians, medical clinics, general hospitals in a secondary healthcare area, and welfare facilities for the elderly requiring long-term care

The results of the sensitivity analysis using variables of full-time and part-time PHNs instead of all PHNs are also shown in Table 4. The significant associations for all causes of death and malignant neoplasms in males found for all PHNs were consistently significant for the relationship with full-time PHNs in the model with full-time and part-time PHNs. A significant positive association between the change of full-time PHNs and female malignant neoplasms was observed in the model of full-time and part-time PHN, though it was not significant in the all PHN model. Another positive association was observed between the changes in part-time PHNs and male cerebrovascular disease in the full-time and part-time PHN models.

The significant associations for all causes of death and malignant neoplasms in males found in the analysis of all PHNs were also confirmed in the stratified analyses by population and EBSMR at baseline (Table 5). In the analysis divided by baseline population, changes in EBSMR were significantly associated with changes in the number of PHNs, for all causes of death in males in both populations below and above 10,000, and for malignant neoplasms in males in populations below 10,000. In the analysis divided by EBSMRs at baseline, there were significant associations between changes in the number of PHNs and changes in EBSMRs when baseline EBSMRs were 100 or more for all causes of death and malignant neoplasms in males. There was also a significant association between change in the number of PHNs and change in EBSMR when the baseline EBSMR was less than 100 for heart disease in females.

**Table 5:** Stratified analysis of the relationship between changes in EBSMRs and PHNs (*n* = 1601)

Variables of change (2015–2010)	**Stratified analysis by baseline population**				**Stratified analysis by baseline EBSMRs**					
	Population < 10,000		Population ≥ 10,000		EBSMR < 100			EBSMR ≥ 100		
	(*n* = 470)		(*n* = 1131)							
	Coef^b^	*P-value*	Coef^b^	*P-value*	(n)	Coef^b^	*P-value*	(n)	Coef^b^	*P-value*
EBSMRs in males^a^										
All causes of death	-1.81	0.009	-1.00	0.026	(780)	0.20	0.638	(821)	-1.90	0.001
Malignant neoplasms	-2.41	0.005	0.02	0.966	(960)	-0.23	0.668	(641)	-1.59	0.015
Heart disease in males	1.61	0.371	-0.38	0.676	(745)	1.16	0.188	(856)	-0.88	0.541
Cerebrovascular disease	-2.18	0.321	-2.21	0.065	(755)	1.09	0.335	(846)	-2.43	0.128
EBSMRs in females^a^										
All causes of death	-0.96	0.309	-0.31	0.536	(844)	-0.95	0.084	(757)	-0.36	0.624
Malignant neoplasms	-0.13	0.906	0.88	0.126	(1120)	0.47	0.442	(481)	-0.30	0.715
Heart disease	-1.83	0.341	-0.91	0.352	(800)	-2.09	0.040	(801)	-0.18	0.909
Cerebrovascular disease	-2.57	0.283	-1.53	0.203	(808)	-0.86	0.443	(793)	-0.52	0.780

*Coef* Coefficient, *EBSMR* Empirical Bayes estimate of standardized mortality ratio, *PHN* Public health nurse

^a^ Dependent variables

^b^ Coefficients of PHNs per 100,000 population (logarithmic transformed) which are adjusted for random effects by prefecture and fixed effects by change of the number of population (logarithmic transformed) and healthcare resources per 100,000 population (2015–2010): physicians, medical clinics, general hospitals in a secondary healthcare area, and welfare facilities for the elderly requiring long-term care

The results of the full Bayesian inference using the BYM model are shown in Table 6. In the model with the number of PHNs at each time point, the higher the number of PHNs, the lower the number of deaths for all causes (estimate, -11.27) and malignant neoplasms (estimate, -14.68) in males [95% credible interval (CI), -19.84 to -2.80, and -25.31 to -3.99, respectively], and for all causes (estimate, -10.43) and malignant neoplasms (estimate, -22.96) in females (95% CI, -19.87 to -0.92, and -35.03 to -11.09, respectively). In the model in which the number of PHNs was divided into the baseline data of 2010 and the change from 2010 to 2015, the baseline results did not differ from the results before the division. For the change in the number of PHNs, the greater the increase in PHNs, the lower the number of deaths for all causes of death (estimate, -22.33) and malignant neoplasms (estimate, -22.46) in males (95% CI, -35.98 to -8.43, and -40.14 to -4.61, respectively), and for all causes of death (estimate, -17.17) in females (95% CI, -32.54 to -2.01).

**Table 6:** Hierarchical Bayesian estimation of the relationship between the number of deaths and PHNs (*n* = 1601)

	**PHNs model**		**Baseline PHNs and change of PHNs model**			
	Number of PHNs^b,c^		Baseline PHNs (2010)^b,c^		Change of PHNs (2015–2010)^b,c^	
	Estimate^d^	(95%CI)	Estimate^d^	(95%CI)	Estimate^d^	(95%CI)
Number of deaths in males^a^						
All causes of death	-11.27	(-19.84 to -2.80)	-13.60	(-22.66 to -4.70)	-22.33	(-35.98 to -8.43)
Malignant neoplasms	-14.68	(-25.31 to -3.99)	-16.42	(-27.51 to -5.21)	-22.46	(-40.14 to -4.61)
Heart disease in males	-9.61	(-26.49 to 7.56)	-12.53	(-30.29 to 5.34)	-22.41	(-50.36 to 5.13)
Cerebrovascular disease	-2.98	(-23.90 to 17.92)	-6.88	(-28.94 to 14.97)	-4.75	(-39.82 to 30.59)
Number of deaths in females^a^						
All causes of death	-10.43	(-19.87 to -0.92)	-13.52	(-23.46 to -3.69)	-17.17	(-32.54 to -2.01)
Malignant neoplasms	-22.96	(-35.03 to -11.09)	-27.20	(-39.32 to -14.92)	-12.94	(-32.61 to 6.51)
Heart disease	-9.43	(-27.65 to 8.98)	-10.17	(-28.98 to 8.70)	-27.35	(-56.94 to 2.21)
Cerebrovascular disease	0.01	(-22.99 to 23.42)	-5.69	(-29.88 to 18.28)	4.21	(-34.39 to 43.11)

*BYM* Besag-York-Mollie, *CI* Credible interval based on quantiles, *ICAR* Intrinsic conditional autoregressive, *PHN* Public health nurse

^a^ Dependent variables

^b^ Per 100,000 population

^c^ Items for which logarithmic transformation is performed

^d^ Estimates are calculated using a no-intercept model, which includes an offset term of the expected number of deaths, random effects by ICAR component for spatial smoothing and component for non-spatial heterogeneity, and fixed effects by population (logarithmic transformed), time (dummy), and the number of healthcare resources per 100.000 population: physicians, medical clinics, general hospitals in a secondary healthcare area, and welfare facilities for the elderly requiring long-term care

Adjustment variables other than time are scaled by 1/1000 because the values of the effects are too small

## Discussion

In this study, the EBSMRs for all causes of death and the three major causes of death trended downward, consistent with the national trends published by the MHLW [29].

The number of PHNs per 100,000 population was higher in small municipalities, which is in line with a previous study [28]. However, the differences in EBSMRs were relatively small and less than medium. For the bivariate relationship between changes in EBSMRs and changes in the number of PHNs per population, the analysis of the baseline population of less than and more than 10,000 showed roughly the same trend as the total population, which provided a certain degree of validity to the combined analysis of all municipalities.

The result of the first-difference model revealed that an increase in the number of PHNs per population was significantly associated with a greater reduction in deaths from all causes and malignant neoplasms in males. Similar associations were shown in the analyses that divided PHNs into full-time and part-time and stratified analyses by population and EBSMRs status at baseline. In the first-difference model of this study, the multiplicity of tests is an issue because four outcomes were tested in the same municipalities in all analyses other than the stratified analysis using the baseline EBSMR. However, the q-values, the adjusted *p*-value controlling for false discovery rate using the Benjamini–Hochberg procedure [37], were less than 0.10 for the significant results except for all causes of death in males in populations above 10,000, generally supporting the results of the first-difference model (see table in Additional file 3: Table S2). Furthermore, the full Bayesian inference using the BYM model confirmed a similar relationship between the number of deaths and changes in the number of PHNs, supporting these results. These sensitivity analyses suggest that the findings of this study are robust.

Some positive associations were found in the full-time and part-time PHN models, but these positive associations were not found in the stratified analysis divided by baseline EBSMR less than 100 and more than 100 (see table in Additional file 4: Table S3). This indicated that the associations were apparent due to confounding by baseline EBSMR.

Although the statistically significant associations of the changes in PHNs were only detected in males in the first-difference model, the analysis by full Bayesian inference showed a relationship between changes in the number of PHNs for all causes of death in males and females and malignant neoplasms in males. Concerning the number of PHNs by full Bayesian inference, there is a similar relationship for all causes of death and malignant neoplasms for males and females, which is consistent with the results of previous studies [18]. However, in this study, the changes in deaths were associated with changes in the number of full-time PHNs, which differed from the results of our previous study that showed associations with the workforce of part-time PHNs [18]. These results were confirmed in the case of a small baseline population and high EBSMR. These findings indicate that in Japan, full-time PHNs may promote improvements through practical activities for municipal health policy challenges such as small population and high EBSMRs, and part-time PHNs may be deployed to maintain improved healthcare conditions such as low EBSMRs in large municipalities. Nevertheless, this study provided further evidence of the impact of municipal PHNs on population health by focusing on changes in longitudinal PHNs and EBSMRs.

This study showed similar results for all causes of death and malignant neoplasms in males. Since malignant neoplasms have been the leading cause of death in Japan for the past 40 years [38], this suggests that a reduction in the number of male deaths from malignant neoplasms due to the workforce of PHNs may lead to a reduction in all causes, resulting in an increase in male life expectancy [39]. Health screening for adults is one of the traditionally important activities of Japanese PHNs [7]. Notably, the number of PHNs is positively associated with the screening rates for some cancers [40], and the cancer screening rates in Japan have increased from 2010 to 2016 [41]. This suggests that the increased PHN workforce may have facilitated the promotion of cancer screening in the community, leading to increased screening rates and consequently affecting mortality from malignant neoplasms. The lack of this effect in females may be explained by the lower cancer screening rates among females than males and the lower age-adjusted mortality rate for malignant neoplasms in females [29].

This study also suggests that the change in PHNs’ workforce may have short-term effects of five years, mainly with regard to malignant neoplasms in males. The time lag between the activities of PHNs and EBSMRs could not be examined in this study due to the absence of comparable data prior to 2010 due to municipal mergers. However, since the effects of activities such as cancer screening are generally captured by five-year survival rates [42], it is reasonable to examine the relationship between PHNs and EBSMRs over a five-year period. In this study, the trend of increasing the number of PHNs at an early stage may have influenced the decrease in EBSMRs over this period, but continued data are needed for a detailed time lag examination.

Cancer is a significant cause of death in many countries, and improving the screening rate has become a global issue [43, 44]. Although few countries have PHNs working as civil servants in every municipality, in Indonesia, the community healthcare system has been improved by developing health personnel like the Japanese PHNs [8]. The effectiveness of PHN activities in depopulated areas and areas with high cancer mortality rates suggests that increasing the workforce of health personnel, such as PHNs in Japan, will be effective in other countries, especially in developing countries.

Stratified analysis by baseline EBSMRs showed a significant negative association between PHNs and EBSMRs in females with a baseline EBSMR of less than 100 for heart disease. This association suggests a different relationship between PHNs’ activity and heart disease than that of malignant neoplasms described above. Concerning female heart disease, PHN activities to address community health issues alone may not be effective, and when EBSMR is low due to the availability of other healthcare resources such as physicians, collaboration with those resources may be effective, but more detailed studies are needed.

A few limitations of this study are that although the fixed-effects model eliminates the effects of factors that are constant over time, it does not adjust for the effects of factors that change over time, such as socioeconomic indicators. Additionally, because the study examines changes over a short period of five years, it may not be possible to examine the long-term effects of the PHN workforce on mortality. Similarly, because of the short period, the analysis was based on a simplified model of the relationship between changes at two-time points. For future research, a more detailed analysis that considers the hierarchy by time is necessary by using longer-term data. Furthermore, this study focuses on the workforce, a structural indicator of activities, and does not examine the actual activities through various process indicators. The use of mortality as an outcome may not reflect the direct effects of the activities of PHNs. For a more detailed understanding of the effects of the PHN workforce, it is necessary to examine the impact on morbidity and physical health and the quality of life and community connections. However, even with such limitations, it is important to clarify from a longitudinal perspective that an increase in the workforce of PHNs effectively influences a decrease in EBSMRs, which can be used as a reference for future decision-making regarding the recruitment of PHNs.

## Conclusions

This study assesses the impact of the workforce of PHNs in municipalities on mortality, which has been underexamined. EBSMR was used to overcome the problem of SMR estimates for small municipalities, and the first-difference model was used for the longitudinal analysis of the two-time points. The results revealed that an increase in the PHN workforce was significantly associated with a greater reduction in deaths from all causes and malignant neoplasms in males, and sensitivity analyses supported this finding. The results of the full Bayesian inference also suggest that the workforce of PHNs may be related to changes in SMR for deaths from all causes in females. This study’s results can be used as a reference for future decision-making regarding the recruitment of PHNs worldwide.

## Supplementary Information

**Additional file 1. **Details on the Besag-York-Mollie (BYM) model and full Bayesian inference methods in this study. The Besag-York-Mollie (BYM) model and the full Bayesian inference method in this study are described in detail, including the model formulation, sampler algorithm, parameter prior distribution, and convergence criteria.

**Additional file 2: Table S1.** All PHN models without random effects by prefecture (n = 1601). Results of analysis using a no-intercept linear model without random effect by the prefecture to compare with the AIC of the model with random effect in Table 4.

**Additional file 3: Table S2.** q-values (the adjusted *p*-value controlling for false discovery rate using the Benjamini–Hochberg procedure). Results for the q-values, which is the adjusted *p*-value controlling for false discovery rate using the Benjamini–Hochberg procedure to examine the issue of multiplicity of tests.

**Additional file 4: Table S3**. Stratified analysis of full-time and part-time PHN model by baseline EBSMRs (*n* = 1601). Results of the stratified analysis, which is divided by EBSMR less than 100 and more than 100 using full-time and part-time PHN models rather than all PHN models.

## Data Availability

The data used in this study are openly available on the portal site for Japanese Government Statistics (e-Stat) at https://www.e-stat.go.jp/. Database names and links to e-Stat for each database (in Japanese, as of March 7, 2023) are listed below. - Population Census (using 2010 and 2015 data).https://www.e-stat.go.jp/stat-search?page=1&toukei=00200521 - Specific Report of Vital Statistics (using FY2008-FY2012 and FY2013-FY2017 data).http://www.e-stat.go.jp/SG1/estat/GL02100104.do?gaid=GL02100102&tocd=00450013 - Report on Regional Public Health Services and Health Promotion Services (using 2010 and 2015 data).http://www.e-stat.go.jp/SG1/estat/NewList.do?tid=000001030884 - Survey of Physicians, Dentists, and Pharmacists (using 2010 and 2014 data).https://www.e-stat.go.jp/stat-search?page=1&toukei=00450026 - Dynamic Survey of Medical Institutions (using 2010 and 2015 data).http://www.e-stat.go.jp/SG1/estat/NewList.do?tid=000001030908 - Survey of Institutions and Establishments for Long-term Care (using 2010 and 2015 data).http://www.e-stat.go.jp/SG1/estat/NewList.do?tid=000001029805

## Abbreviations

AIC: Akaike's information criterion
BYM: Besag-York-Mollie
CDR: Crude death rate
CI: Credible interval (based on quantiles)
EB: Empirical Bayes
EBSMR: Empirical Bayes estimate of standardized mortality ratio
ICAR: Intrinsic conditional autoregressive
MHLW: Ministry of Health, Labour and Welfare
PHN: Public health nurse
SMR: Standardized mortality ratio
VIF: Variance inflation factor
