# Supplementary material for: Relationship between changes in the public health nurses’ workforce and the empirical Bayes estimates of standardized mortality ratio: a longitudinal ecological study of municipalities in Japan
Source: BMC Health Serv Res. 2023 Mar 17;23:266. doi: 10.1186/s12913-023-09273-2 (PMC10022064; doi:10.1186/s12913-023-09273-2)
Supplement: Supplementary file 1 — Additional file 1. Details on the Besag-York-Mollie (BYM) model and full Bayesian inference methods in this study. The Besag-York-Mollie (BYM) model and the full Bayesian inference method in this study are described in detail, including the model formulation, sampler algorithm, parameter prior distribution, and convergence criteria. [file 12913_2023_9273_MOESM1_ESM.docx]

**Additional file 1.**

**Details on the Besag-York-Mollie (BYM) model and full Bayesian inference methods in this study**

In this study, the full Bayesian inference for the BYM model^1-3^ was performed by Stan,^4^ a probabilistic programming platform performing full Bayesian inference using Hamiltonian Monte Carlo (HMC), following Morris et al.^3^ Stan’s No U-Turn Sampler (NUTS) using HMC provides better and more robust estimates for models such as the BYM model that have complex posteriors than samplers that use Gibbs or Metropolis algorithms.^5^

The BYM model in this study can be described as follows.

$o_{i} \sim\mathrm{Poisson}\left( \mu_{i} \right)$

$\log\left( \mu_{i} \right)=\log\left( e_{i} \right)+\boldsymbol{x}_{i}\beta+\varphi_{i}+\epsilon_{i}$

$\varphi_{i} | \varphi_{i\neq j} \sim N \left( \bar{\varphi}_{i}, {\sigma_{\varphi}^{2}}/{m_{i}} \right)$

$$\epsilon_{i} \sim\left( 0, \sigma_{\epsilon}^{2} \right),$$

where

1. $o$ is the observed number of deaths in each municipality.
2. $e$ is the number of deaths expected from the age-specific reference mortality rates. The standardized mortality ratio (SMR) of each municipality is $o/e$.
3. $\mu$ is the expected value of $o$, the parameter of the Poisson distribution: $\mu_{i}=e_{i}\theta_{i}$, where $\theta_{i}$ is the expected value of the SMR, and the $\log\left( e_{i} \right)$ is treated as an offset term.^6^
4. $\boldsymbol{x}$ is the matrix of independent explanatory variables; hence, $\boldsymbol{x}_{i}$ is the vector of independent variables for municipality $i$, and $\beta$ is vector of regression coefficients which are constant across all regions, that is, fixed effects.
5. $\varphi$ is an intrinsic conditional autoregressive (ICAR) component for special smoothing, that is, structured random effects. For the adjacency of municipalities, municipalities bordering each other are defined as neighboring municipalities, and neighboring islands are determined by referring to significant shipping routes. Each $\varphi$ is normally distributed with a mean equal to $\bar{\varphi}$, the average of its neighboring municipalities. In the analysis by Stan, $\varphi$ was estimated by the following pairwise difference formula, following Morris et al.^3^

$p\left( \varphi\right) \propto\exp\left( -\frac{1}{2}\sum_{i<j} \left( \varphi_{i}-\varphi_{j} \right)^{2} \right).$

1. m is the number of neighboring municipalities of municipality $i$.
2. $\epsilon$ is a component for non-spatial heterogeneity, that is, unstructured random effects. Each $\epsilon$ is normally distributed with a mean equal to 0.

The standard BYM model usually includes a fixed intercept term corresponding to the expected value of the overall risk level. However, our model did not include the intercept term because the relative risk in this study is SMR, calculated based on the national value aggregated from all municipalities in 2010; as a result, the expected value of the overall risk level corresponding to the intercept term is estimated to be 1, which becomes 0 when logarithmically transformed. The independent variables consist of the number of public health nurses (PHNs), physicians, medical clinics, general hospitals in a secondary health care area, welfare facilities for the elderly requiring long-term care (per 100,000 population, respectively), population, and a time dummy variable equal to 0 for 2010 and 1 for 2015 representing the change over time of the dependent variable. As in the first-difference model, logarithmic transformations were performed for the number of PHNs and population. In addition, for the PHN variable, we first examined the relationship between the number of PHNs and the number of deaths using data from both time points, and then to examine the impact of changes in the number of PHNs on this relationship, we performed a similar analysis with the PHN variable split into the baseline number of PHNs in 2010 and the change in the number of PHNs from 2010 to 2015.

We used inverse gamma distribution $\Gamma^{-1}$(0.5, 0.0005) for the prior distributions of the parameters $\sigma_{\varphi}$ and $\sigma_{\epsilon}$, referring to Lawson et al.^7^ and Nakatani,^2^ and uniform distributions for the other parameters. The estimation of ICAR component φ by Morris et al.^3^ also uses a soft sum-to-zero constraint that the mean of φ in each spatially connected region follows a distribution of N (0, 0.001) to make φ identifiable. Since the municipalities at the two time points are not spatially connected, we applied the soft sum-to-zero constraint for each time point.

To fit the BYM model to the municipality data using Stan, we ran 4 chains of 8000 iterations with a thinning value of 1, where the first 1000 steps were the warm-up phase, and the last 7000 steps were the sampling phase. The chains were considered convergent when the R-hat statistic was less than 1.1, and the effective sample size (ESS) was greater than 100 for all parameters.^8^ In the actual analysis, the independent variables other than time were scaled by 1/1000 because the values of regression coefficients were too small for the parameters to converge.

**References**

1. Besag J, York J, Mollié A. Bayesian image restoration, with two applications in spatial statistics. Ann Inst Stat Math. 1991;43:1-20.

2. Nakaya T. Evaluating socio-economic inequalities in cancer mortality by using areal statistics in Japan: A note on the relation between municipal cancer mortality and areal deprivation index. [in Japanese]. Proceedings of the Institute of Statistical Mathematics. 2011;59:239-265.

3. Morris M, Wheeler-Martin K, Simpson D, Mooney SJ, Gelman A, DiMaggio C. Bayesian hierarchical spatial models: implementing the Besag York Mollié Model in stan. Spat Spatiotemporal Epidemiol. 2019;31:100301.Stan Development Team.

4. Stan User’s Guide and Reference Manual. version 2.29. http://mc-stan.org/; Accessed February 22, 2022; 2022.

5. Hoffman MD, Gelman A. The no-u-turn sampler: adaptively setting path lengths in Hamiltonian Monte Carlo. J Mach Learn Res. 2014;15:1593-1623.

6. Lawson AB, Browne WJ, Vidal Rodeiro CL. Disease Mapping with WinBUGS and MLwiN. New York: John Wiley & Sons, Inc; 2003.

7. Lawson AB. Bayesian Disease Mapping: Hierarchical Modeling in Spatial Epidemiology. 3rd ed. New York: Chapman & Hall/CRC; 2018.

8. Matsuura K. Bayesian Statistical Modeling Using Stan and R. Wonderful R Series, Volume 2. [in Japanese]. Tokyo: Kyoritsu Shuppan; 2016.
